# The importance of vegetation density for tourists’ wildlife viewing experience and satisfaction in African savannah ecosystems

**DOI:** 10.1371/journal.pone.0185793

**Published:** 2017-09-28

**Authors:** Ugo Arbieu, Claudia Grünewald, Matthias Schleuning, Katrin Böhning-Gaese

**Affiliations:** 1 Senckenberg Biodiversity and Climate Research Centre (SBiK-F), Frankfurt am Main, Germany; 2 Department of Biological Sciences, Goethe Universität, Frankfurt am Main, Germany; University of Pretoria, SOUTH AFRICA

## Abstract

Southern African protected areas (PAs) harbour a great diversity of animals, which represent a large potential for wildlife tourism. In this region, global change is expected to result in vegetation changes, such as bush encroachment and increases in vegetation density. However, little is known on the influence of vegetation structure on wildlife tourists’ wildlife viewing experience and satisfaction. In this study, we collected data on vegetation structure and perceived mammal densities along 196 road transects (each 5 km long) and conducted a social survey with 651 questionnaires across four PAs in three Southern African countries. Our objectives were 1) to assess visitors’ attitude towards vegetation, 2) to test the influence of perceived mammal density and vegetation structure on the easiness to spot animals, and 3) on visitors’ satisfaction during their visit to PAs. Using a Boosted Regression Tree procedure, we found mostly negative non-linear relationships between vegetation density and wildlife tourists’ experience, and positive relationships between perceived mammal densities and wildlife tourists’ experience. In particular, wildlife tourists disliked road transects with high estimates of vegetation density. Similarly, the easiness to spot animals dropped at thresholds of high vegetation density and at perceived mammal densities lower than 46 individuals per road transect. Finally, tourists’ satisfaction declined linearly with vegetation density and dropped at mammal densities smaller than 26 individuals per transect. Our results suggest that vegetation density has important impacts on tourists’ wildlife viewing experience and satisfaction. Hence, the management of PAs in savannah landscapes should consider how tourists perceive these landscapes and their mammal diversity in order to maintain and develop a sustainable wildlife tourism.

## Introduction

Cultural ecosystem services are increasingly studied to identify how humans derive cultural values and benefits from nature [1]. Wildlife tourism is a cultural ecosystem service where people directly interact with nature through outdoor recreational activities [2]. Numbers of wildlife tourists are constantly rising in protected areas (PAs) worldwide [3], generating valuable revenues for local and national economies [4] and contributing to biodiversity conservation efforts [5–7]. To ensure sustainable management of PAs, a good understanding of visitors appreciation of PAs, and their satisfaction related to their wildlife viewing experience is crucial [8–10].

In Africa, wildlife tourism relies mostly on the observation of free-ranging animals [8,11,12]. Consequently, visitors’ satisfaction is expected to be associated with animal observations [13]. In particular, charismatic animals such as predators and large ungulates [11,12,14] are strongly sought after by wildlife tourists [15]. Thus, because of the economic challenges at stake in PAs and the implications for conservation, it is fundamental that PA management maintains the potential to observe these iconic species in their natural habitat [16].

The communities of large ungulates and predators, preferred by wildlife tourists, are in general found in tropical savannah ecosystems [17,18]. Vegetation composition and structure of tropical savannahs depend on a dynamic equilibrium between grass and woody plants [19,20] that is influenced, among other factors, by water availability, soil conditions, herbivory, and fire frequency [21–23]. Under current environmental conditions, grasses are often dominant over woody species, providing an open vegetation structure [19]. This open vegetation structure is an essential asset for wildlife tourism because it allows good observations of wild animals [16].

Global change is expected to affect savannah ecosystems in African PAs. Increases in CO_2_ atmospheric concentrations and changes in rainfall could alter the dominance of grass species over woody species [19,22] with long lasting and potentially irreversible effects on vegetation composition and structure [22,24,25]. Woody encroachment [26] occurs when the woody cover and vegetation density increases, causing substantial changes in savannah ecosystems [22,24,27,28].

Bush encroachment can have direct effects on mammal communities, and also indirect effects on wildlife tourism. Previous work has shown that bush encroachment could lead to changes in mammal community structure and composition, owing to animals’ feeding adaptations [23]. From a wildlife tourism perspective, wildlife visitors are likely to experience more difficulties in observing animals, due to reduced visibility [16]. Even though vegetation contributes to the nature experience through enjoyment of landscape aesthetics [29], dense vegetation could be perceived negatively by PA visitors. This could ultimately lead to a lower degree of visitors’ satisfaction in densely vegetated areas. Although many studies have described bush encroachment and potential ecological impacts [26,30], notably on mammal communities [31], there is little empirical evidence on how it will affect wildlife viewing activities and visitor satisfaction (but see [16]).

To understand how vegetation is mediating wildlife tourists’ experience during their visits to PAs, we collected data on vegetation structure, perceived mammal densities and visitors’ satisfaction in four PAs across South Africa, Botswana and Namibia. These four PAs cover a large rainfall gradient, and reflect a diversity of savannah types, from open grasslands to densely vegetated areas with high woody plant cover. To assess visitor’s attitudes towards vegetation and their satisfaction with spotting animals and wildlife viewing we conducted a survey based on questionnaires in the four PAs. Our objectives were 1) to quantify the visitors’ attitudes towards vegetation in the PAs by asking whether vegetation contributed negatively or positively to their experience, 2) to test if mammal densities and vegetation had an influence on the easiness to spot animals, and 3) to quantify the effects of mammal densities and vegetation on visitors’ satisfaction during their visit to PAs.

## Methods

### Study area

**Fig 1:**
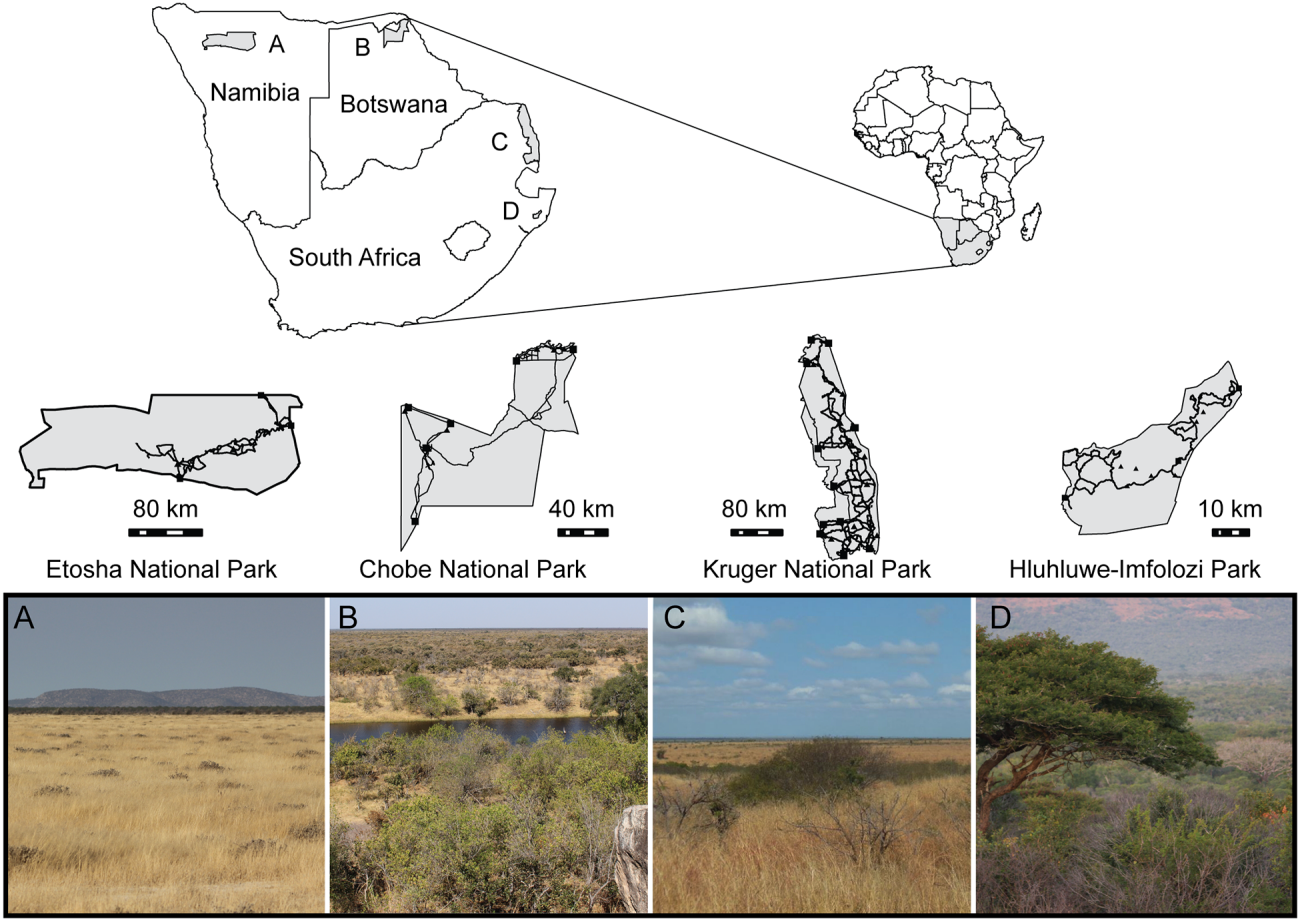
Map of study area. Map of the Southern African region and details of the four protected areas where we collected data on vegetation structure, perceived mammal densities and conducted the social surveys.

We collected data on vegetation structure, visibility, perceived mammal abundance and visitors’ attitudes and satisfaction in four PAs, namely Etosha National Park (Namibia; in the following Etosha), Chobe National Park (Botswana; Chobe), Kruger National Park (South Africa; Kruger) and Hluhluwe-Imfolozi Park (South Africa; Hluhluwe-Imfolozi) (Fig 1). Permission to conduct ecological and social surveys were granted by the Ministry of Environment and Tourism of Namibia (Etosha National Park), the Ministry of Environment, Wildlife and Tourism of Botswana (Chobe National Park), SANParks (Kruger National Park) and Ezemvelo KZN Wildlife (Hluhluwe-Imfolozi Park). Participation in social surveys was voluntary. The anonymity of all participants was guaranteed during data collection and analysis (formal consent was therefore not necessary). All data were collected during the dry season, which is the recommended period for visiting the parks. Data collection was conducted in Etosha in October 2014, in Chobe from June to July 2014, in Kruger from June to August 2012, and in Hluhluwe in May 2014. These four PAs are all located in southern Africa and, despite differences in their mammal communities, herbivore and predator communities in each PA result from the same regional species pool. The PAs offer a variety of savannah landscapes, from open grasslands to areas with more woody vegetation, reflecting a large rainfall gradient from the west to the east of the region (Table 1).

**Table 1:** Protected area characteristics. IUCN categories for Protected Areas were retrieved from Protected Planet (https://www.protectedplanet.net/). N transects = number of transects distributed in each Protected Area; N visitors = the total number of visitors in respective years; N respondents = number of respondents in our social surveys.

Protected area	IUCN category	Area (km^2^)	Mean rainfall (mm/y)	Vegetation description	N visitors (year)	N transects	N respondents
Etosha National Park	II—National Park	22.270	320–450	Arid savannah, salt pan surrounded by grasslands and mixed savannah (*Colophospermum mopane*, *Acacia nebrownii*) [32,33]	200.000 (2014)	50	153
Chobe National Park	Ib—Wilderness Area	10.700	550–700	Riparian woodlands (*Croton megalobotrys*, *Capparis tomentosa*, *Combretum mossambicense*) and dry scattered grasslands with mixed woodlands (*Colophospermum mopane*, *Baikiaea plurijuga*, *Combretum* spp.) [34–36]	240.000 (2013)	40	158
Kruger National Park	II—National Park	18.992	500–700	Plain grasslands interspersed with woody vegetation *(Acacia*, *Combretum*, *Sclerocarya* and *Colophospermum* spp.) [37]	1.400.000 (2010)	78	204
Hluhluwe-Imfolozi Park	Not reported—nature reserve managed by Ezemvelo KZN Wildlife	900	635–990	Savannah woodland with varying amount of woody cover (*Acacia* spp.) [38]	140.000 (2014)	28	136

### Vegetation structure

In order to assess visitors’ attitudes towards vegetation in relation to the actual vegetation structure in each of the four PAs, we collected data on vegetation structure along 5 km touristic road transects. These transects were distributed over the existing road network of each PA, under the condition that they were accessible to the wildlife tourists, and that they covered as much of the rainfall gradient as possible in each PA. We distributed 50, 40, 78 and 28 road transects which were 5 km long (n = 196 in total) along the public roads of Etosha, Chobe, Kruger and Hluhluwe, respectively. We extracted the mean annual rainfall for each transect from the WorldClim database (http://www.worldclim.org, v 1.4, 30 seconds resolution), using 500 m buffers around each transect in ArcGIS (v. 10.1).

We estimated the structure of the vegetation placed directly next to the roads, to reflect as best as possible the visitors’ perceptions of vegetation within PAs. Thus, we estimated vegetation structure over squares of 20 m x 20 m on each side of the road at each kilometre of the transect (km 0 [start of the transect], km 1, km 2, km 3, km 4 and km 5 [end of the transect]). We took two different measures of vegetation structure. First, we distinguished between seven vegetation types, burned areas [referred to as ‘burned’ in the analysis]; rocky areas [‘rock’]; bare ground [‘bare ground’]; short grass, *i*.*e*. grass smaller than 50 cm [‘short grass’]; tall grass, *i*.*e*. grass taller than 50 cm [‘tall grass’]; shrubs, *i*.*e*. woody plants that were smaller than 3 m [‘shrubs’]; and trees, *i*.*e*. woody plants that were taller than 3 m). We estimated the percentage of each vegetation type in each square at each kilometre along the transect. Values of the seven vegetation types sum up to 100%. We calculated the average vegetation cover for each vegetation type per transect by taking the arithmetic mean over the 12 values (six points on each side of the road). Second, we collected data on the vegetation height structure by estimating the percentage of vegetation corresponding to each of three height categories, ‘short’ (vegetation smaller than 50 cm), ‘intermediate’ (vegetation comprised between 50 cm and 3m) and ‘tall’ (vegetation taller than 3 m). Values of the three vegetation heights also sum up to 100%. We calculated the average of each vegetation height per transect by taking the arithmetic mean over the 12 values. Several vegetation variables were closely correlated (see S1 Table) and the number of variables was therefore reduced with a PCA prior to the analyses (see below).

We collected data on visibility, to quantify the ability to detect medium-sized animals from a wildlife tourist’s perspective, adopting methods commonly used in distance sampling [39]. We estimated the perpendicular distance at which we would be able to identify a fictitious adult warthog (*Phacochoerus africanus*), to the left and to the right side of the road at every kilometre of a transect, using a laser range finder. The warthog is used to estimate visibility because it is small in stature and its body mass lies at the median of the ungulate species usually studied (ca. 82 kg) [39]. We derived a single estimate of visibility per transect by taking the arithmetic mean over the 12 values.

### Perceived mammal densities

To investigate how vegetation could affect the observation of large mammals within PAs, we estimated probabilities of sighting large mammals along the road transects. We adopted this probabilistic approach to reflect *perceived* mammal densities from the visitors’ perspective, rather than *true* mammal densities within PAs. We replicated counts of all herbivores and predators (see S2 Table in Supporting Information for a list of species) along the road transects within PAs, and complemented our predator counts with sightings from visitors (see Supporting Information, S1 Methods).

For each species of ungulate, we derived an estimate of perceived density per transect by taking the arithmetic mean of the number of individuals observed over the three replicates. For each species of predator, we derived an estimate of perceived density by calculating the arithmetic mean of the number of individuals observed (by us and visitors) over the number of times a transect had been driven (by us and the visitors). Finally, for each transect, the total mammal density is the sum over the density of each species. This measure consequently reflects the mammal density perceived by tourists along each studied transect.

### Questionnaires

To quantify visitors' attitude towards vegetation, how vegetation mediates the easiness to spot animals and which factors determine satisfaction of wildlife tourists in the four PAs, we used questionnaires organized in four sections: (1) details of the visit, (2) driving route details and personal preferences towards animals, (3) predator sightings information and (4) attitudes towards vegetation structure and landscape (see the questionnaire in Supporting Information, S2 Methods). The sampling population was restricted to visitors or groups of visitors over 18 years old, randomly selected in public areas (camping grounds, picnic sites) at lunch time and in the evenings. We collected visitor characteristics related to their country of residence (international vs. local), group size, duration of stay, number of previous visits to the PA and the type of visit (private vs. guided tour) (section 1). We could not collect further social characteristics because in most cases, questionnaires were answered by groups of people, and not individuals. The duration of each questionnaire was on average 10 to 15 minutes.

To understand wildlife tourists’ attitude towards vegetation, we asked them if “in their opinion, vegetation contributed positively or negatively to their enjoyment on that day” (section 4, question g.; answer yes or no). Likewise, to assess the easiness to spot animals while driving in the PA, we asked if “it was easy to spot animals” (section 4, question a., answer: yes or no). To quantify the respondents’ level of satisfaction, we asked them if “their expectations had been met” during their drives in the PA (section 2, question II. d.). This question was asked after a series of questions related to the choices made for the specific day, and the expectations associated with it. Consequently, this satisfaction level represents the overall satisfaction they had obtained during the drive of the specific day. We used a Likert scale ranging from 1 (not satisfied) to 10 (entirely satisfied).

### Statistical analysis

All statistical analyses were done with R 3.1.1 software (R Core Team 2014) and dedicated packages. In the first part of the analysis, we related vegetation structure to rainfall, visibility and mammal densities at the level of the 196 road transects. To reduce the seven categories of vegetation types and the three categories of vegetation heights into its main orthogonal components, a Principal Component Analysis (PCA) on the correlation matrix of the variables was performed (prcomp’ function, ‘stats’ package). To test the relationships between the main vegetation components (PC1 and PC2) and rainfall, visibility and mammal densities across the transects and PAs, we fitted these three variables onto the PC biplot (‘envfit’ function, ‘vegan’ package). In the later analysis involving Boosted Regression Trees, we focussed on the two main vegetation components derived from the PCA (PC1 and PC2) to avoid collinearity among predictor variables (S1 Table).

In the second part of the analysis, we related visitors’ attitudes towards the vegetation, easiness to spot animals and satisfaction to vegetation structure and mammal densities. We did not include visibility as an independent predictor as it was closely correlated to the main vegetation gradient (Pearson correlation between visibility and PC1, r = 0.83, n = 612; S1 Table). We fitted alternative models including shrub cover and grass cover instead of PC1 and PC2, respectively. These analyses were conducted at the level of the 651 questionnaires, taking into account the route the respondents had driven on the specific day. To this end, we took the average of the vegetation variables, PC1, PC2 and mammal density values over the transects passed by the visitors during the specific day. To model the influence of these variables on visitors’ attitudes towards vegetation, easiness to spot animals, and satisfaction, we used a Boosted Regression Trees (BRT) modelling technique and the gbm.step routine (‘gbm’ package) described by Elith *et al*. [40]. BRT is a model building rather than a model testing approach, which is very suitable if little is known on the relationships between response and predictor variables [40]. We tested three separate models of attitudes towards vegetation, easiness to sport animals and visitors’ satisfaction. The first model included the attitude towards vegetation as a binary response variable (1 meaning positive attitude, 0 negative attitude), and PC1, PC2 and PA identity as predictor variables. The second model included the easiness to spot animals as the response variable (1 meaning easy spotting, 0 meaning not easy spotting), and mammal density, PC1, PC2, and PA identity as predictor variables. The third model included the satisfaction score as the response variable (ranging from 1 to 10), and mammal density, PC1, PC2, and PA identity as predictor variables. We repeated the same procedure and replaced PC1 and PC2 by shrub and grass cover, respectively.

BRT models were optimized by adjusting tree complexity (tc), learning rate (lr) and bag fraction (bf) and running different combinations of these three parameters (tc = 1-2-3; lr = 0.01–0.005–0.001–0.0005; bf = 0.5–0.75). We used cross-validation deviance to identify the best model (lowest deviance indicates the best model). We used the percent of explained deviance to assess the performance of the models, calculated as: 1-(residual deviance/total deviance). We derived the contributions of each predictor to the respective model from BRT. In all BRT models, we quantified thresholds in the non-linear predictions of the relationship between response and predictor variables using the ‘segmented’ function (‘segmented’ package).

## Results

### Vegetation structure across transects and protected areas

**Fig 2:**
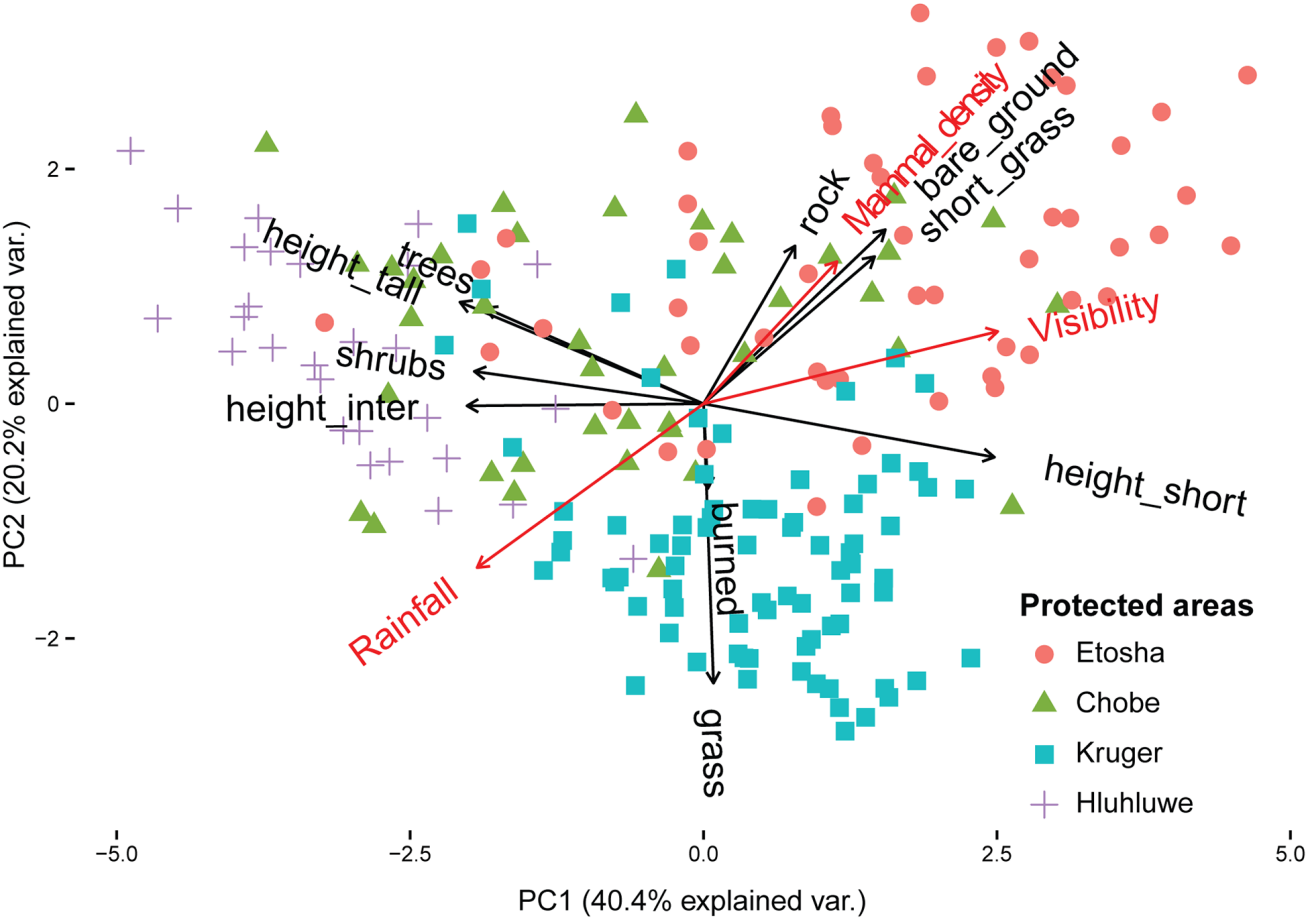
Principal Component Analysis (PCA) plot of the vegetation structure encountered along 196 road transects in four protected areas of Southern Africa. Black vectors represent seven variables of vegetation types (burned, rock, bare ground, short grass, tall grass–‘grass’–, shrubs, trees), and three variables of vegetation heights (short–‘height_short’–, intermediate–‘height_inter’, tall–‘height_tall’–). Red vectors (rainfall, visibility and perceived mammal densities) represent variables plotted on top of the ordination plot in order to show correlations with vegetation structure. The smaller the angle between two vectors on the plot, the higher the correlation between them.

The first principal component (PC1) and the second principal component (PC2) accounted for 60.6% of the variability in the vegetation variables (Fig 2). PC1 represented a gradient from closed vegetation types (negative PC1 values) to open and short vegetation (positive PC1 values), and was mostly correlated with shrub cover (S1 Table). PC2 described a gradient from grasslands (negative PC2 values) to more complex vegetation types including woody vegetation (positive PC2 values) and was mostly correlated with grass cover (see Supporting Information, S1 and S3 Tables). The PC scores describe a continuous vegetation gradient across the four PAs (Fig 2). Hluhluwe-Imfolozi transects were located in areas with the densest woody vegetation, while Etosha transects had the most open vegetation. Chobe and southern Kruger transects were placed between these two PAs with Kruger located primarily in grassland habitats.

Rainfall, visibility and mammal densities correlated significantly with the two first PCA components (P < 0.01 in all cases). Higher rainfall was correlated with lower values of PC1 and PC2, *i*.*e*. woody vegetation types and grasslands. In contrast, higher visibility and mammal densities were related to transects with higher values of PC1 and PC2 (i.e. open habitats and shorter vegetation, see Fig 2).

### Attitude of wildlife tourists towards vegetation

We collected in total 153, 158, 204 and 136 questionnaires in Etosha, Chobe, Kruger and Hluhluwe-Imfolozi, respectively. The social characteristics of respondents varied substantially across PAs (see S4 Table). The proportion of international visitors (from 20.1% in Kruger up to 85.0% in Etosha), the number of previous visits to PAs (2.8 previous visits on average in Chobe up to 33.4 in Kruger) as well as the average group size or length of stay (only 1.9 days on average in Hluhluwe-Imfolozi and up to 5.6 in Kruger) were quite heterogeneous among respondents (see S4 Table). The majority of the respondents to our questionnaires used private vehicles to drive in the four PAs. Some respondents were unable to give the details of their route, and were removed from the analyses, resulting in 143, 151, 186 and 132 exploitable questionnaires in Etosha, Chobe, Kruger and Hluhluwe-Imfolozi, respectively.

**Fig 3:**
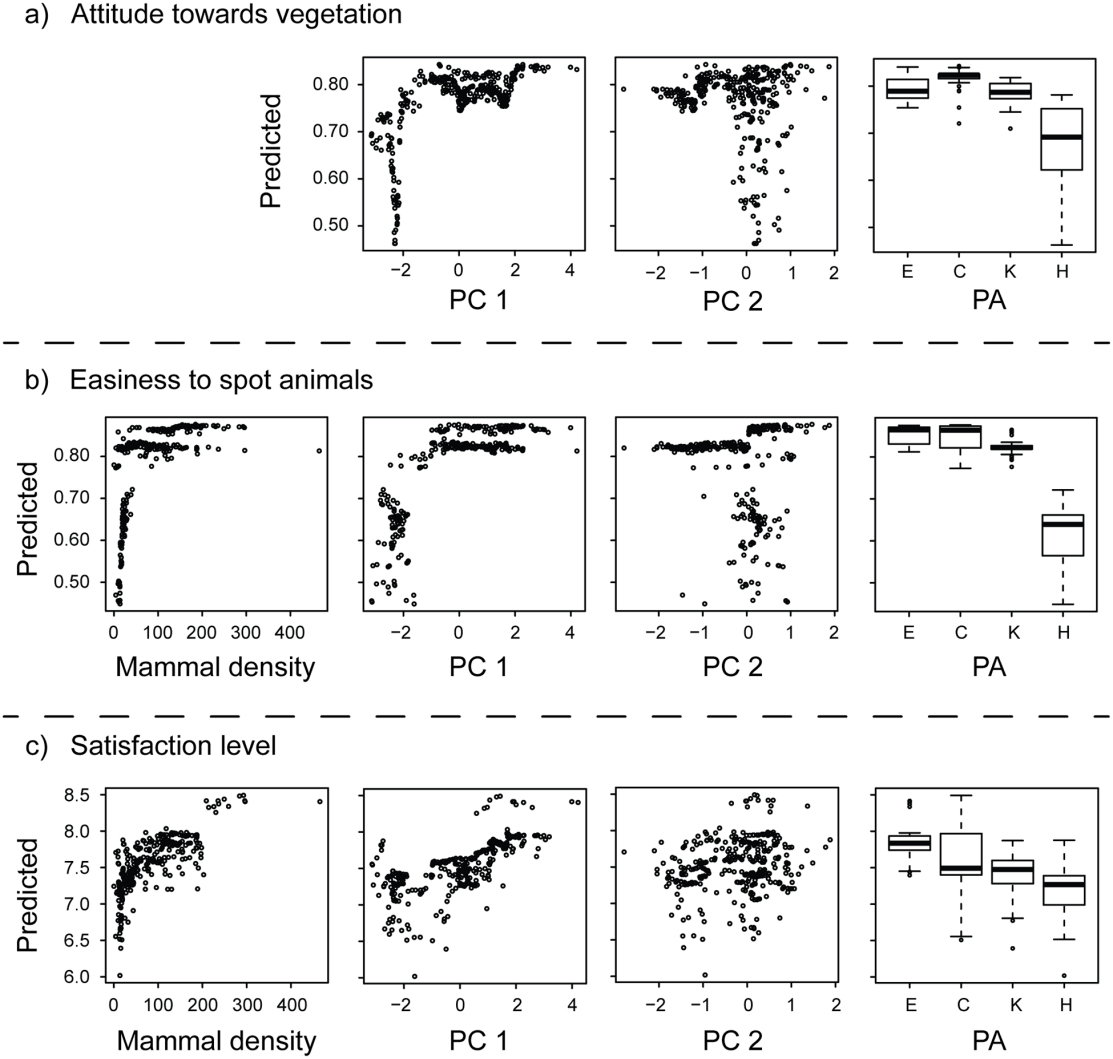
Results from Boosted Regression Trees (BRT) analyses of a) wildlife tourists’ attitudes towards vegetation, b) easiness to spot animals and c) wildlife tourists’ satisfaction levels in four protected areas. The plots show the fitted values predicted by each BRT model. Predicted values range between 0 and 1 for visitors’ attitudes towards vegetation (0 = negative; 1 = positive) and easiness to spot animals (0 = not easy; 1 = easy) (binomial models) and from 0 to 10 for the satisfaction level (0 = not satisfied; 10 = fully satisfied) (Gaussian model). PC1-2 = principal components 1–2 from PCA on vegetation variables (see Fig 2); PA = protected area (E = Etosha, C = Chobe, K = Kruger, H = Hluhluwe-Imfolozi); Mammal density = perceived mammal densities along road transects.

Of the 597 respondents, 461 (77%) reported that vegetation contributed positively and 136 (23%) that vegetation contributed negatively to their enjoyment in the PA on the specific day. Wildlife tourists’ attitudes towards vegetation were mostly related to PC1 (relative contribution, 70%) and, to a lesser extent, to PC2 (23%) and PA (7%) (Table 2). Tall and dense woody vegetation with high proportions of shrubs were perceived negatively by wildlife tourists (Fig 3a) if the vegetation density in the transects passed a threshold of very closed vegetation, corresponding to a PC1 score of -1.09 (Table 2). We found similar results if we tested shrub and grass cover instead of PC1 and PC2 (S1 Fig). Shrub cover was the most important predictor (47%) compared to grass and PA predictors and was perceived negatively if shrub cover exceeded 32% (S5 Table).

**Table 2:** Results of Boosted Regression Trees models showing the influence of each predictor on the respective response variable, threshold estimates in non-linear relationships, model parameters and model performance. PC1-2 = principal components 1–2 (accounting for > 60% of the variance) from a Principal Component Analysis on vegetation variables (see Methods); PA = protected area; Mammals = perceived mammal densities along road transects; tc = tree complexity; lr = learning rate; bf = bag fraction; CV-deviance = Cross-Validated deviance; Performance = 1 –(residual deviance / total deviance).

Model	Predictor	Influence	Threshold	Conf. Interval	tc	lr	bf	CV-deviance (SE)	Performance
**Attitude**	PC1	69.53	-1.09	[-1.33;-0.84]	3	0.01	0.5	1.04 (0.01)	0.07
	PC2	23.31	0.27	[0.07;0.47]					
	PA	7.16							
**Easiness**	Mammals	24.18	46.21	[41.82;50.61]	2	0.01	0.75	0.96 (0.02)	0.11
	PC1	22.95	-0.6	[-0.77;-0.42]					
	PC2	12.99	-0.05	[-0.24;0.13]					
	PA	39.88							
**Satisfaction**	Mammals	43.87	26.86	[23.45;30.27]	2	0.01	0.75	5.08 (0.28)	0.08
	PC1	36.73	-1.64	[-1.84;-1.44]					
	PC2	18.07	0.48	[0.07;0.90]					
	PA	1.33							

### Easiness to spot animals

Of the 609 respondents who answered this question, 482 respondents (79%) answered that spotting animals was easy, while 127 (21%) found it difficult. PA had the highest influence on the ease to spot animals (relative contribution, 40%), while mammal densities and PC1 had an almost equal influence in the model (24 and 23%, respectively; see Table 2). Spotting animals tended to be more difficult in Hluhluwe-Imfolozi than in the three other PAs (Fig 3b). The relationships between easiness to spot animals and mammal densities and vegetation structure were non-linear. Notably, the easiness to spot animals dropped steeply at values of mammal densities lower than 46 individuals per transect and at PC1 values lower than -0.60 (Fig 3b and Table 2). In the model including shrub and grass cover instead of PC1 and PC2, PA and mammal densities were more important for the easiness to spot mammals than shrub and grass cover (S1 Fig and S5 Table). Similar to the model of tourists' attitudes towards vegetation, the easiness to spot mammals dropped at a shrub cover larger than 31% (S5 Table).

### Wildlife tourists’ satisfaction

Among all questionnaires, 9 respondents did not provide a satisfaction scores. Average satisfaction score across the four PAs was 7.5 on a scale from 0 to 10 (standard deviation of 2.3). Mammal density (relative contribution, 44%) and PC1 (37%) were the most influential factors in the BRT model (Table 2). The relationship between satisfaction and mammal densities was non-linear, with a threshold identified at 26 individuals per transect below which satisfaction dropped markedly (Fig 3c and Table 2). Tourists’ satisfaction and PC1 were positively related, corresponding to lower satisfaction scores in dense vegetation (Fig 3c). PC2 (18%) and PAs (1%) did not have a strong influence on satisfaction scores. In the models with shrub and grass cover replacing PC1 and PC2, we found similar patterns (S1 Fig and S5 Table). A shrub cover larger than 43% resulted in a reduced tourist satisfaction (S5 Table).

## Discussion

Our results demonstrate that relationships between vegetation structure, perceived mammal densities and tourists’ wildlife viewing experience and satisfaction were non-linear, suggesting that minor changes in habitat structure or mammal densities can have a great impact on wildlife tourists’ experiences. In particular, wildlife tourists’ attitudes towards vegetation were negative in areas with dense woody vegetation, while tourists’ ease to spot mammals and their satisfaction dropped at low mammal densities and decreased with increasing vegetation density.

### Attitude of wildlife tourists towards vegetation

The attitude of wildlife tourists towards vegetation was overall positive across the four PAs. Indeed, 77% of the respondents reported that vegetation was contributing positively to their safari enjoyment on the specific day. Previous studies have highlighted the importance of natural environments and landscape aesthetics in contributing to human well-being [29,41,42]. In our study, however, the vegetation openness had a strong non-linear relationship with visitors’ attitudes (Fig 3a). Consequently, it seems that visitors are positive towards vegetation, up to a threshold of about 30% of shrub cover above which it was perceived negatively. This pattern is notably reflected by the high variance of predicted values in Hluhluwe-Imfolozi, which is the PA where vegetation density was the highest. Interestingly, some negative attitudes were also formulated in PAs where the vegetation was more open. In Etosha, for instance, the monotony of the landscape at this time of year (October) was perceived negatively. Nevertheless, the model of visitors’ attitudes towards vegetation was mainly explained by vegetation ‘openness’. This finding notably concurs with a previous study claiming that savannah landscapes, *i*.*e*. open plains with few large trees, were aesthetically more pleasing than areas that are more densely vegetated [16].

### Wildlife viewing, perceived mammal densities and vegetation

Spotting animals was considered easy by the majority of wildlife tourists across all PAs. A variety of large herbivore species (12 species in Etosha, 16 in Chobe, 17 in Kruger and 13 in Hluhluwe-Imfolozi) and all larger predators were observed during our transect counts in the four PAs (except wild dog in Etosha). Nevertheless, 23% of the respondents noted that spotting of wildlife was not easy. First, the ease to spot animals was considerably reduced at low levels of mammal densities because mammals were more difficult to spot along these transects. In particular, tourists found it difficult to spot large mammals if the densities were lower than 46 individuals per 5 km transect. Second, vegetation significantly reduced the ease to spot animals when exceeding a threshold of vegetation density at about 30% of shrub cover. Increased vegetation density and taller vegetation had a direct negative impact on wildlife tourists’ visibility, as shown by the close correlation between visibility and PC1 (Fig 2). Bush encroachment is currently modifying savannah landscapes and woody species grow taller than grass species, creating densely vegetated, closed habitats [27]. This could impair visitors’ wildlife experience in a PA dominated by closed savannah habitats. In fact, PA was the most influential predictor for the easiness to spot animals (Table 2). Hluhluwe-Imfolozi was the PA with the highest vegetation density, corresponding to the highest difficulty of spotting wildlife across the four PAs [16]. The relative difficulty to spot animals is part of the wildlife experience of tourists. Nevertheless, when mammal densities and vegetation structure reach a certain threshold, wildlife viewing experience is affected and appears to become too difficult for wildlife tourists.

### Satisfaction, perceived mammal densities and vegetation

Overall, wildlife tourists visiting the four PAs expressed a relatively high level of satisfaction regarding their wildlife experience across the four PAs. Mammal densities and vegetation openness had a strong influence on their satisfaction level. Mammal densities affected satisfaction level in a non-linear manner, with satisfaction dropping at a threshold of about 26 mammal individuals per transect (Table 2). Thus, tourists’ satisfaction dropped at values of mammal densities lower than that found in the analysis of the easiness to spot mammals. This shows that tourists tolerated low mammal densities even if affecting their sighting probabilities, but only to a certain extent. Below this threshold, low mammal densities were indeed related to a substantial reduction in the tourists’ satisfaction. In addition, tourists’ satisfaction declined linearly with increasing vegetation density. Thus, changes in vegetation resulting in progressive closing of savannah landscapes may negatively impact wildlife tourists’ satisfaction.

Overall, the BRT model highlights an important link between wildlife tourists’ satisfaction and their perceived environment both in terms of large mammals and vegetation. Tourists’ satisfaction is an important indicator of PA attractiveness and can have implications for PA management. The social survey performed in Hluhluwe-Imfolozi by Gray & Bond [16] suggests that the level of satisfaction is linked to the wildlife viewing success. Consequently, they infer that a substantial part of the PA economy could be affected by lower number of revisits due to decreased satisfaction, especially in the case of international wildlife tourists [16]. In our BRT model, the PA factor had a very low influence on the overall satisfaction level, meaning that the relationships between satisfaction and environmental factors are consistent across the four PAs. Consequently, the satisfaction of wildlife tourists is expected to be influenced by vegetation and mammal densities not only in Hluhluwe-Imfolozi, but equally in other PAs. Hence, areas with dense vegetation and low probabilities of observing large mammals are expected to be less satisfying for wildlife tourists in savannah ecosystems, which could have deleterious economic effects on PAs’ economies.

### Implications and recommendations for management

Our result shed light on the role of vegetation structure in modulating wildlife observations in four PAs in Southern Africa. On the one hand, wildlife tourists were generally satisfied with their safari experience, while, on the other hand, our models showed that thresholds in perceived mammal densities and vegetation densities exist, below which wildlife viewing experience and satisfaction appear to be impaired. Our study was performed in a variety of savannah landscapes and therefore covers a large vegetation gradient across Southern Africa. Ongoing climate change is expected to enhance bush encroachment in the Southern African region [19,22]. Our results suggest that increased vegetation density could alter the wildlife visitors’ ability to observe wildlife. The repercussions of bush encroachment on nature-based tourism remain poorly studied, but we provide evidence that increased vegetation density may indeed decrease visitors’ satisfaction and PA attractiveness. Since tourists are highly adaptable [43,44], they could switch destinations as a result of decreased attractiveness. Thus, management measures should be guided towards maintaining relative openness of vegetation within PAs and savannah ecosystems (*i*.*e*. open grasslands with reduced tree cover), to satisfy ecological needs of the majority of large mammals occurring in these PAs, and to optimize wildlife tourists’ experience. Understanding relationships between wildlife tourism and ecosystems is crucial for sustainable PA management and, in the long run, for the conservation of ecosystems and their iconic mammal species. In that respect, the non-linear patterns and thresholds we identified in the responses of wildlife tourists to the environment improve our understanding of the complexity of cultural ecosystem services.

## Supporting information

S1 Methods Description of perceived mammal densities. (PDF)

S2 Methods Questionnaires used for the social survey. (PDF)

S1 Fig Results from alternative Boosted Regression Trees (BRT) analyses of a) wildlife tourists’ attitudes towards vegetation, b) easiness to spot animals and c) wildlife tourists’ satisfaction levels in four protected areas including shrub cover and grass cover as predictors. The plots show the fitted values predicted by each BRT model. Predicted values range between 0 and 1 for visitors’ attitudes towards vegetation (0 = negative; 1 = positive) and easiness to spot animals (0 = not easy; 1 = easy) (binomial models) and from 0 to 10 for the satisfaction level (0 = not satisfied; 10 = fully satisfied) (Gaussian model). Shrubs = percentage of shrub cover; grass = percentage of grass cover (taller than 50 cm); PA = protected area (E = Etosha, C = Chobe, K = Kruger, H = Hluhluwe-Imfolozi); Mammal density = perceived mammal densities along road transects.
(PDF)

S1 Table Correlation matrix between potential predictor variables for the Boosted Regression Tree models. h_short = short vegetation height category; h_intert = intermediate vegetation height category; h_tall = high vegetation height category; mammals = mammal density perceived by tourists; PC1-2 = principal components 1–2 (accounting for > 60% of the variance) from the Principal Component Analysis on vegetation variables.
(PDF)

S2 Table List of large mammal species recorded during transect counts. List of all larger mammal species of predators and ungulates recorded during road transects in Etosha, Chobe, Kruger National Parks and Hluhluwe-Imfolozi Game Reserve. Common and scientific names are based on the Atlas of Mammals of Africa (volumes V & VI, Kingdon & Hoffmann 2013).
(PDF)

S3 Table Principal Component Analysis loadings. Loadings of the variables on each of the two first components of the Principal Component Analysis performed on the seven vegetation types and three vegetation heights (‘height_short’, ‘height_inter’ and ‘height_tall’ stand for short, intermediate and tall vegetation respectively). PC1 represents a measure of vegetation openness and PC2 represents the transition in vegetation from grasslands to woodlands.
(PDF)

S4 Table Social characteristics of visitors in the four protected areas. Origin (international vs. local, *i*.*e*. residents of Southern African countries), group size, length of stay, first timer (i.e. percentage of people who visit a park for the first time), number of previous visits and the type of the visit (private tour vs. guided tour) are presented. Numbers displayed are averages, with standard deviation in brackets. Percentages that do not sum up to 100 are the result of missing data from visitors who could not give an answer to the question.
(PDF)

S5 Table Results of alternative Boosted Regression Trees models including shrub and grass variables instead of PC1 and PC2. PA = protected area; Mammals = perceived mammal densities along road transects; tc = tree complexity; lr = learning rate; bf = bag fraction; CV-deviance = Cross-Validated deviance; Performance = 1 –(residual deviance / total deviance).
(PDF)
