# Supplementary material for: The importance of vegetation density for tourists’ wildlife viewing experience and satisfaction in African savannah ecosystems
Source: PLoS One. 2017 Sep 28;12(9):e0185793. doi: 10.1371/journal.pone.0185793 (PMC5619831; doi:10.1371/journal.pone.0185793)
Supplement: S1 Methods — (PDF) [file pone.0185793.s001.pdf]

S1 Methods

To estimate the potential for observing mammal species in the different protected areas (PAs), we counted all larger mammal species (ungulates and predators) encountered along the transects while driving at slow speed (ca. 15 km/h) in a four-wheel drive truck. Two observers scanned the area either on the right or on the left side of the road. When an animal or a group of animals was spotted, we stopped and counted individuals. We replicated transect counts three times, with at least three full days between temporal replicates. Because predators are difficult to observe in the wild, we estimated predator abundance by complementing our own sightings with visitors' sightings from questionnaires (see below, section 3 of the questionnaires). By merging the data collected with these two approaches, we provide a better estimate of predator densities along the transects. We only considered lion (*Panthera leo*), leopard (*Panthera pardus*), spotted hyena (*Crocuta crocuta*), cheetah (*Acinonyx jubatus*) and wild dog (*Lycaon pictus*) as predators, because they are the largest and most regularly observed predators in these PAs. We collected data on predators whenever we could observe them along the roads of each PA (during transect counts, or while we were commuting within PAs). We identified the species, counted individuals, and located the sighting with a GPS-device. For visitors' predator observations, visitors were asked about the predator identity, number of individuals and the location of the observation. We further asked visitors to draw on a map the route they had been taking on the day of observation, to document which of the transects they had been driving through (section 2, question a of the questionnaire, see below). We used common maps provided by each PA, because they are the ones used by visitors for orientation. When respondents were unsure about the identity of predators, the location of the sighting, or the routes they had taken in the PA, we did not retain the information for the analyses. We digitized all predator sighting locations in a geographic information system (ARCGIS v. 10.1), and retained only the ones falling within the transects for abundance estimates.
