# Supplementary material for: The importance of vegetation density for tourists’ wildlife viewing experience and satisfaction in African savannah ecosystems
Source: PLoS One. 2017 Sep 28;12(9):e0185793. doi: 10.1371/journal.pone.0185793 (PMC5619831; doi:10.1371/journal.pone.0185793)
Supplement: S2 Methods — (PDF) [file pone.0185793.s002.pdf]

S2 Methods

Date__________________ Time__________________ Interview/Map ID______________

Location: ____

Interviewer:____

Interview language:____

SECTION I: Visit details

a. Where are you from? ____

b. For how long are you staying in the park? ____

c. Is it your first stay in the Park?____

d. If not, how many times have you been here before? ______________________________

e. At which Camp are you staying? ____

f. Have you been staying in this Camp all the time or have you been staying elsewhere in the park on this trip?

____

g. Accommodation type (camping, hut, lodge etc.)? _____________________________

h. Are you travelling on your own or in a group? _____________________________

i. Is your trip self-organized or by a tour operator? _____________________________

j. What is the main reason(s) behind your visit to this Park (not more than 3)?

1.__________________________________

2.__________________________________

3.__________________________________

SECTION II: DRIVING ROUTE AND PERSONAL PREFERENCES

I. <u>Did you do a (game) drive today in the park?</u>

a. <u>If no</u>: What else did you do? ____

Was there any reason for doing so? ____________________________________

<u>b. If yes</u>: Did you go by yourself or did you join a guided tour? (maybe time/ start of tour/name of guide) ____

c. For how long have you been on the (game) drive?

____

II. <u>Where in the park did you go to?</u>

a. Can you please show on this map **(provided by interviewer)** where you went? Please mark/indicate approximately the route (if possible).

b. Was there any particular reason for going to this area in the park or for booking this game drive? ____________________________________

c. Which were your topmost expectations (not more than 3) for that day?

1.____________________________________

2.____________________________________

3.____________________________________

d. Have your expectations been met? (On a scale from 1 to 10, 1 being not met at all, 10 being absolutely met) ______________________________________

e. Can you rank the following park features by order of importance (from 1-5)?

Landscape uniqueness _________________________

Wildlife viewing _________________________

Calm and quietness _________________________

Luxury of some accommodations ___________

Botany _________________________

f. Are there any park features missing that you think are of high interest? Which ones? ____

g. Could you rank the following animal groups by order of spotting preference (from 1 to 6)?

- Birds __________________________________
- Reptiles __________________________________
- Insects __________________________________
- Small mammals (meerkat, mongoose, ground squirrel...) ___
- Ungulates (gemsbok, springbok, eland…) _______________
- Big cats ____

h. Are there any animal groups missing that you think are of high interest? Which ones? ____

i. For your route in the park today have you had any expectations which animals you would see in that area of the park? _______________________________

j. Which animals did you hope to see/did you want to see?

____

SECTION III: PREDATOR SIGHTINGS

a. Did you see one of the following predators (give number, and locate on the map)?

	Lion	Cheetah	Leopard	Spotted Hyena	Wild dog
Number					
Distance					
Number					
Distance					

At what distance from the car/road (suggest categories)?

a) directly next to car b) less than 200 m c) 500 m d) more than 500 m

SECTION IV: VEGETATION

a. Was it easy to spot animals?

Why yes? ____

Why not? ____

b. Had vegetation density an influence on this? How?

____

c. How would you describe vegetation density? (1-10, 1 being very dense/thick vegetation, 10 being open landscape) _________________________________

d. How would you describe visibility? (1-10)´ _______________________________

e. What would you say was the main vegetation type/landscape on your drive today?

(*e.g.* mainly bush/grass/tree; tree-grass mix etc.) _________________________________

f. What did you expect vegetation/visibility/landscape to be like prior to this drive? Was this expectation met? ____

g. In your opinion – did vegetation contribute positively or negatively to your enjoyment today? Why positively, why negatively?_________________________

h. In general - what kind of vegetation/landscape in the park do you enjoy most? ____

i. Which type makes game viewing most enjoyable? _________________________
