# Supplementary material for: The importance of vegetation density for tourists’ wildlife viewing experience and satisfaction in African savannah ecosystems
Source: PLoS One. 2017 Sep 28;12(9):e0185793. doi: 10.1371/journal.pone.0185793 (PMC5619831; doi:10.1371/journal.pone.0185793)
Supplement: S1 Fig — The plots show the fitted values predicted by each BRT model. Predicted values range between 0 and 1 for visitors’ attitudes towards vegetation (0 = negative; 1 = positive) and easiness to spot animals (0 = not easy; 1 = easy) (binomial models) and from 0 to 10 for the satisfaction level (0 = not satisfied; 10 = fully satisfied) (Gaussian model). Shrubs = percentage of shrub cover; grass = percentage of grass cover (taller than 50 cm); PA = protected area (E = Etosha, C = Chobe, K = Kruger, H = Hluhluwe-Imfolozi); Mammal density = perceived mammal densities along road transects. (PDF) [file pone.0185793.s003.pdf]

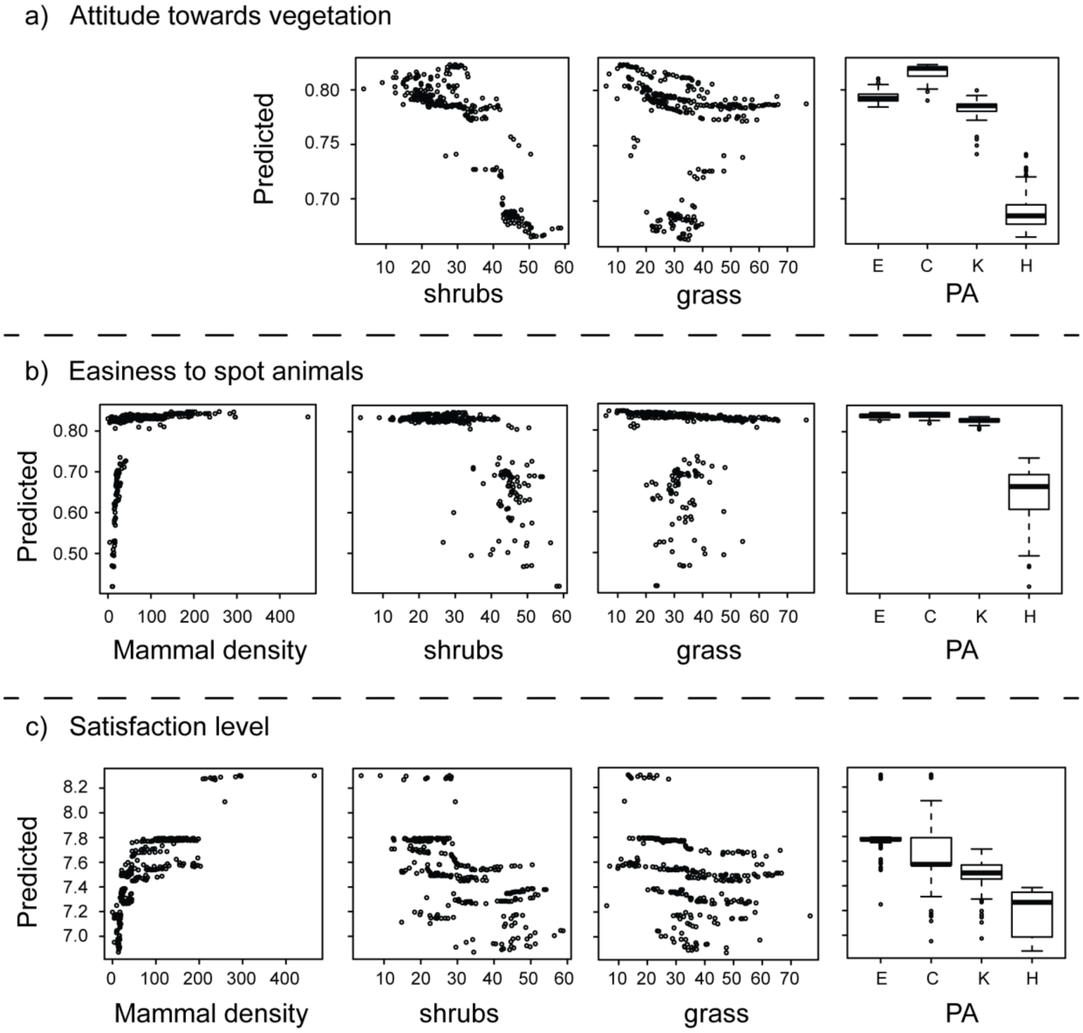

S1 Fig. Results from alternative boosted regression trees (BRT) analyses of a) wildlife tourists' attitudes towards vegetation, b) easiness to spot animals and c) wildlife tourists' satisfaction levels in four protected areas including shrub cover and grass cover as predictors. The plots show the fitted values predicted by each BRT model. Predicted values range between 0 and 1 for visitors' attitudes towards vegetation (0 = negative; 1 = positive) and easiness to spot animals (0 = not easy; 1 = easy) (binomial models) and from 0 to 10 for the satisfaction level (0 = not satisfied; 10 = fully satisfied) (Gaussian model). Shrubs = percentage of shrub cover; grass = percentage of grass cover (taller than 50 cm); PA = protected area (E = Etosha, C = Chobe, K = Kruger, H = Hluhluwe-Imfolozi); Mammal density = perceived mammal densities along road transects.
