# Supplementary material for: The importance of vegetation density for tourists’ wildlife viewing experience and satisfaction in African savannah ecosystems
Source: PLoS One. 2017 Sep 28;12(9):e0185793. doi: 10.1371/journal.pone.0185793 (PMC5619831; doi:10.1371/journal.pone.0185793)
Supplement: S1 Table — h_short = short vegetation height category; h_intert = intermediate vegetation height category; h_tall = high vegetation height category; mammals = mammal density perceived by tourists; PC1-2 = principal components 1–2 (accounting for > 60% of the variance) from the Principal Component Analysis on vegetation variables. (PDF) [file pone.0185793.s004.pdf]

S1 Table. Correlation matrix between potential predictor variables for the Boosted Regression Tree models. h_short = short vegetation height category; h_intert = intermediate vegetation height category; h_tall = high vegetation height category; mammals = mammal density perceived by tourists; PC1-2 = principal components 1-2 (accounting for > 60% of the variance) from the Principal Component Analysis on vegetation variables.

	visibility	bare_ground	short_grass	rock	burned	grass	shrubs	trees	h_short	h_inter	h_tall	PC1	PC2	mammals
visibility	1													
bare_ground	**0.70**	1												
short_grass	0.39	0.43	1											
rock	0.48	0.48	0.16	1										
burned	-0.09	-0.17	-0.21	-0.13	1									
grass	-0.02	-0.57	-0.56	-0.21	0.19	1								
shrubs	**-0.73**	-0.50	-0.55	-0.37	-0.12	-0.07	1							
trees	**-0.69**	-0.31	-0.32	-0.14	-0.06	-0.29	0.54	1						
h_short	**0.76**	0.49	0.56	0.12	0.04	0.12	**-0.85**	**-0.81**	1					
h_inter	**-0.68**	-0.50	-0.57	-0.05	0.01	-0.01	**0.85**	0.55	**-0.93**	1				
h_tall	**-0.72**	-0.39	-0.44	-0.17	-0.09	-0.21	**0.69**	**0.95**	**-0.90**	**0.67**	1			
PC1	**0.83**	**0.67**	**0.69**	0.33	-0.05	-0.12	**-0.87**	**-0.75**	**0.95**	**-0.88**	**-0.85**	1		
PC2	-0.08	**0.51**	0.21	0.30	-0.29	**-0.89**	0.32	0.45	-0.36	0.27	0.41	-0.13	1	
mammals	0.39	**0.60**	**0.73**	0.29	-0.20	**-0.66**	-0.35	-0.20	0.40	-0.41	-0.32	0.57	0.49	1
