# Supplementary material for: The importance of vegetation density for tourists’ wildlife viewing experience and satisfaction in African savannah ecosystems
Source: PLoS One. 2017 Sep 28;12(9):e0185793. doi: 10.1371/journal.pone.0185793 (PMC5619831; doi:10.1371/journal.pone.0185793)
Supplement: S2 Table — List of all larger mammal species of predators and ungulates recorded during road transects in Etosha, Chobe, Kruger National Parks and Hluhluwe-Imfolozi Game Reserve. Common and scientific names are based on the Atlas of Mammals of Africa (volumes V & VI, Kingdon & Hoffmann 2013). (PDF) [file pone.0185793.s005.pdf]

S2 Table. List of large mammal species recorded during transect counts. List of all larger mammal species of predators and ungulates recorded during road transects in Etosha, Chobe, Kruger National Parks and Hluhluwe-Imfolozi Game Reserve. Common and scientific names are based on the Atlas of Mammals of Africa (volumes V & VI, Kingdon & Hoffmann 2013).

English name	Scientific name	Etosha	Chobe	Kruger	Hluhluwe-Imfolozi
**African lion**	*Panthera leo*	X	X	X	X
**African leopard**	*Panthera pardus*	X	X	X	X
**Cheetah**	*Acinonyx jubatus*	X	X	X	X
**Spotted hyena**	*Crocuta crocuta*	X	X	X	X
**Wild dog**	*Lycaon pictus*	-	X	X	X
**African elephant**	*Loxodonta africana*	X	X	X	X
**African buffalo**	*Syncerus caffer*	-	X	X	X
**Black rhinoceros**	*Diceros bicornis*	X	-	-	-
**Blue wildebeest**	*Connochaetes taurinus*	X	X	X	X
**Bushbuck**	*Tragelaphus scriptus*	-	-	X	X
**Common hippopotamus**	*Hippopotamus amphibius*	-	X	*	-
**Common duiker**	*Sylvicapra grimmia*	-	-	X	X
**Common warthog**	*Phacochoerus africanus*	X	X	X	X

**Gemsbok**	*Oryx gazella*	X	-	-	-
**Giraffe**	*Giraffa camelopardalis*	X	X	X	X
**Greater kudu**	*Tragelaphus strepsiceros*	X	X	X	X
**Impala**	*Aepyceros melampus*	X	X	X	X
**Klipspringer**	*Oreotragus oreotragus*	-	-	X	-
**Mountain reedbuck**	*Redunca fulvorufula*	-	-	X	-
**Nyala**	*Tragelaphus angasii*	-	-	-	X
**Plains zebra**	*Equus quagga*	X	X	X	X
**Puku**	*Kobus vardonii*	-	X	-	-
**Red duiker**	*Cephalophus natalensis*	-	-	-	X
**Red hartebeest**	*Alcelaphus buselaphus*	X	-	-	-
**Red lechwe**	*Kobus leche*	-	X	-	-
**Roan antelope**	*Hippotragus equinus*	-	X	-	-
**Sable antelope**	*Hippotragus niger*	-	X	X	-
**Southern reedbuck**	*Redunca arundinum*	-	-	X	-
**Springbok**	*Antidorcas marsupialis*	X	-	-	-

**Steenbok**	*Raphicerus campestris*	X	X	X	-
**Tsessebe**	*Damaliscus lunatus*	X	-	-	-
**Waterbuck**	*Kobus ellipsiprymnus*	-	X	X	-
**White rhinoceros**	*Ceratotherium simum*	-	-	X	X

*Hippos were excluded in Kruger for methodological reasons.
