# Supplementary material for: The importance of vegetation density for tourists’ wildlife viewing experience and satisfaction in African savannah ecosystems
Source: PLoS One. 2017 Sep 28;12(9):e0185793. doi: 10.1371/journal.pone.0185793 (PMC5619831; doi:10.1371/journal.pone.0185793)
Supplement: S3 Table — Loadings of the variables on each of the two first components of the Principal Component Analysis performed on the seven vegetation types and three vegetation heights (‘height_short’, ‘height_inter’ and ‘height_tall’ stand for short, intermediate and tall vegetation respectively). PC1 represents a measure of vegetation openness and PC2 represents the transition in vegetation from grasslands to woodlands. (PDF) [file pone.0185793.s006.pdf]

**S3 Table. Principal Component Analysis loadings.** Loadings of the variables on each of the two first components of the Principal Component Analysis performed on the seven vegetation types and three vegetation heights (‘height_short’, ‘height_inter’ and ‘height_tall’ stand for short, intermediate and tall vegetation respectively). PC1 represents a measure of vegetation openness and PC2 represents the transition in vegetation from grasslands to woodlands.

| | PC1 | PC2 |
|---|---|---|
| Bare ground | 0.30 | 0.40 |
| Short grass | 0.28 | 0.34 |
| Rock | 0.15 | 0.37 |
| Burned | 0.01 | -0.20 |
| Grass | 0.02 | -0.65 |
| Shrubs | -0.38 | 0.08 |
| Trees | -0.36 | 0.22 |
| Height_short | 0.48 | -0.12 |
| Height_inter | -0.39 | -0.00 |
| Height_tall | -0.40 | 0.24 |
