# Supplementary material for: The importance of vegetation density for tourists’ wildlife viewing experience and satisfaction in African savannah ecosystems
Source: PLoS One. 2017 Sep 28;12(9):e0185793. doi: 10.1371/journal.pone.0185793 (PMC5619831; doi:10.1371/journal.pone.0185793)
Supplement: S4 Table — Origin (international vs. local, i.e. residents of Southern African countries), group size, length of stay, first timer (i.e. percentage of people who visit a park for the first time), number of previous visits and the type of the visit (private tour vs. guided tour) are presented. Numbers displayed are averages, with standard deviation in brackets. Percentages that do not sum up to 100 are the result of missing data from visitors who could not give an answer to the question. (PDF) [file pone.0185793.s007.pdf]

S4 Table. Social characteristics of visitors in the four Protected Areas. Origin (international vs. local, *i.e.* residents of Southern African countries), group size, length of stay, first timer (i.e. percentage of people who visit a park for the first time), number of previous visits and the type of the visit (private tour vs. guided tour) are presented. Numbers displayed are averages, with standard deviation in brackets. Percentages that do not sum up to 100 are the result of missing data from visitors who could not give an answer to the question.

	Origin (%)		Group size	Length of stay (days)	First timer (%)		N of previous visits	Type of visit (%)	
	International	Local			yes	no		private	guided
Etosha	85.0	15.0	4.3 (4.6)	3.7 (5.0)	75.8	24.2	10.8 (20.5)	81.7	18.3
Chobe	58.2	41.8	7.5 (7.0)	3.4 (2.2)	70.3	29.7	2.8 (3.0)	58.2	41.8
Kruger	20.1	79.9	2.5 (1.3)	5.6 (5.1)	16.2	82.8	33.4 (37.8)	92.6	5.7
Hluhluwe	38.2	61.8	7.2 (21.3)	1.9 (1.6)	35.3	64.0	25.5 (30.2)	81.6	16.9
