# Supplementary material for: The importance of vegetation density for tourists’ wildlife viewing experience and satisfaction in African savannah ecosystems
Source: PLoS One. 2017 Sep 28;12(9):e0185793. doi: 10.1371/journal.pone.0185793 (PMC5619831; doi:10.1371/journal.pone.0185793)
Supplement: S5 Table — PA = protected area; Mammals = perceived mammal densities along road transects; tc = tree complexity; lr = learning rate; bf = bag fraction; CV-deviance = Cross-Validated deviance; Performance = 1 –(residual deviance / total deviance). (PDF) [file pone.0185793.s008.pdf]

S5 Table. Results of alternative Boosted Regression Trees models including shrub and grass variables instead of PC1 and PC2. PA = protected area; Mammals = perceived mammal densities along road transects; tc = tree complexity; lr = learning rate; bf = bag fraction; CV-deviance = Cross-Validated deviance; Performance = 1 – (residual deviance / total deviance).

Model	Predictor	Influence	Threshold	Conf. Interval	tc	lr	bf	CV-deviance (SE)	Performance
**Attitude**	shrub	47.44	31.59	[30.78; 32.4]	1	0.001	0.5	1.06 (0.01)	0.03
	grass	16.06	32.52	[30.62; 34.42]					
	PA	36.50							
**Easiness**	Mammals	28.53	45.86	[41.48; 50.25]	2	0.005	0.75	0.96 (0.02)	0.09
	shrub	11.41	31.24	[29.70; 32.78]					
	grass	6.96	33.34	[30.69; 35.99]					
	PA	53.10							
**Satisfaction**	Mammals	67.71	30.53	[27.77; 33.30]	1	0.001	0.75	5.10 (0.28)	0.04
	shrub	22.81	42.72	[38.35; 47.1]					
	grass	8.81	37.82	[33.57; 42.07]					
	PA	0.66							
